# Return to Sport after Adolescent Idiopathic Scoliosis (AIS) Correction Surgery: A Retrospective Data Analysis

**DOI:** 10.3390/jcm12041551

**Published:** 2023-02-16

**Authors:** Wojciech Pepke, Abhilash Madathinakam, Tom Bruckner, Tobias Renkawitz, Stefan Hemmer, Michael Akbar

**Affiliations:** 1 Department of Orthopaedics, Heidelberg University Hospital, 69118 Heidelberg, Germany; 2 Institute of Medical Biometry and Informatics, University of Heidelberg, 69120 Heidelberg, Germany; 3 Meoclinic, Friedrichstraße 71, 10117 Berlin, Germany

**Keywords:** adolescent idiopathic scoliosis, AIS, correction surgery, Cobb angle, back to sport

## Abstract

Sports are relevant to younger populations in society. Adolescent idiopathic scoliosis (AIS) patients who undergo surgical correction of the spine are often intensively involved in sports. For that, returning to the sport is often an important concern for the patients and their families. To the best of our knowledge, there is still a lack of scientific data indicating established recommendations about the time of returning to sport activities after surgical spinal correction. The aim of this study was to investigate (1) when AIS patients return to athletic activities after a posterior fusion, and (2) if they change their activities postoperatively. Furthermore, another question was (3) if the length of the performed posterior fusion or (4) fusion to the lower lumbar spine could have an influence on the rates or time of returning to sport activities postoperatively. Data collection was performed using questionnaires assessing patients’ contentment and athletic activity. Athletic activities were categorized into three categories: (1) contact, (2) contact/non-contact and (3) non-contact sports. The intensity of exercised sports, the time of returning to the sport and changes in sport habits were documented. Radiographs were evaluated pre- and postoperatively to determine the Cobb angle and the length of the posterior fusion via the identification of the upper (UIV) and lower instrumented vertebra (LIV). Stratification analysis due to the fusion length was performed to answer a hypothetical question. This retrospective survery of 113 AIS patients treated with a posterior fusion revealed that, on average, returning to sport activities required 8 months of postoperative rest. The preoperative to postoperative rate of patients participating in sport activities increased from 88 (78%) to 94 (89%). Furthermore, postoperatively, a relevant shift of exercised activities from contact to non-contact sports was noted. Further subanalysis revealed that only 33 subjects were able to return to exactly the same athletic activities as before surgery (10 months postoperatively). The assessment of radiographs revealed that in this study group, the length of the performed posterior fusion and fusions to the lower lumbar spine had no influence on the time of return to athletic activities. The results of this study might shed some light on postoperative recommendations for sport activities after AIS treatment with a posterior fusion and may be beneficial for surgeons treating patients.

## 1. Introduction

Adolescent idiopathic scoliosis (AIS) is a complex three-dimensional deformity of the spine that deteriorates the curvature in the frontal plane, thoracic kyphosis in the sagittal plane and axial vertebral rotation in the transverse plane. These skeletal changes lead to an alteration in the back shape. If left untreated, AIS may contribute to degenerative changes that lead to debilitating pain, the loss of spinal mobility, loss of function and disability. Depending on the time of the onset of scoliosis, cardiac and pulmonary dysfunction may also accompany these symptoms [1].

The management of idiopathic scoliosis is based on established treatment protocols [2,3,4]. Due to the severity of AIS, the therapy modalities are non-operative (physio therapeutics, brace therapy) or surgical. With regard to surgical interventions, a large multitude and variety have been described in the literature [5]. These include different surgical procedures (anterior, posterior or combined) and different types of implants for correction. Within the last 20 years, the sophistication of spinal implants has increased rapidly. In contrast to the initial surgical therapy of scoliosis with, for e.g., Harrington instrumentation, introduced in the 1960s [6], modern procedures such as a multi-segmental fusion allow for the early mobilization of patients without the need for postoperative casts or braces. Several studies have been published comparing different approaches to the spine (anterior, posterior or combined) and different implant systems [2,7,8,9,10]. A significant variety of implants and approaches for surgical treatment of the spine are available, but double-rod posterior instrumentation seems to be the preferred surgical procedure in the case of scoliosis progression.

Surgical treatment decisions raise many questions for patients [11]. Consequently, a well-founded consultation between the patient and spine surgeon is needed in order to address the concerns of the patient. In particular, surgical procedures which involve a spinal fusion are often linked in the patient’s mind with the lack of capacity to pursue sporting activities [12,13]. A study of long-term sports activities of surgically treated AIS patients is needed in order to answer patients’ concerns about their prospects in regard to returning to athletics postoperatively. There is still a lack of scientific data indicating established recommendations about the time of returning to sport activities after surgical spinal correction. The current guidelines for postoperative participation in sports are based on experts’ opinions as there are no evidence-based recommendations regarding rehabilitation after surgery [14]. Whereas one author recommends returning to non-contact athletic activities 6 months and returning to collision sports 1 year after surgery, other surgeons do not recommend exercising in any contact sports after the surgical procedure [15]. For that, no detailed and specific recommendations on the postoperative ability to return to athletic activities after a spinal fusion can be given at this time. On the other hand, within the past few years, the first study groups have published studies about the resumption of sport activities after a spinal fusion in AIS patients. In their review of five retrospective studies and one prospective study, Barile et al. reported about the safety of returning to any sport activities, even those with extreme spinal loads that occur while exercising (e.g., gymnastics or golf) [16]. In their retrospective survey of 95 AIS patients after a posterior fusion, another group reported that surgically treated AIS patients returned to athletics much earlier than expected [17]. Interestingly, after six months, 54% had returned to non-contact and 63% had returned to contact sports [17]. In contrast, they could not recognize any patient or curve characteristics (Lenke type, LIV) that were associated with a delay in sport resumption [17]. On the other hand, Ruffilli et al. recognized in their survey of 112 AIS patients that a younger age, higher Lenke type and less severe main curve predicted a faster return to sport activities [18]. In our opinion, further investigations are needed to establish common recommendations in terms of the postoperative timepoint of returning to sport activities. To the best of our knowledge, our study includes the highest number of study cohorts compared to similar studies. Furthermore, in the current study, the stratification of the fusion length, analysis of postoperative pain relief and changes of pain frequency might be contributory in understanding behavioral patterns associated with the return to sport activities.

The aim of this study was (1) to investigate when AIS patients after a posterior fusion return to athletic activities and (2) if they change their activities postoperatively. We hypothesized that a longer fusion or a fusion to the lower lumbar spine might be associated with worse rates or a later timepoint of returning to athletic activities. To investigate that, we further aimed (3) to determine if the length of the performed posterior fusion or the fusion to the lower lumbar spine (4) correlates with the time of returning to sports.

## 2. Materials and Methods

### 2.1. Study Cohort

**Figure 1:**
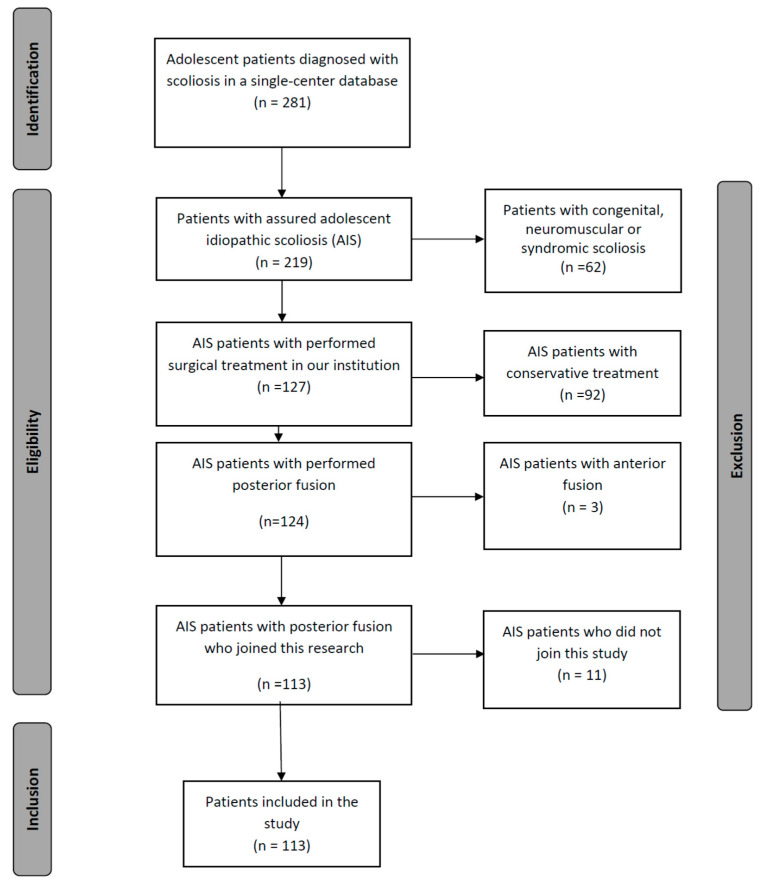
Flow diagram describing the inclusion process of the study population.

In this retrospective single-center cohort study, surgically treated AIS patients who had begun therapy between 2010 and 2018 were included. All patients were treated with the posterior fusion procedure by the two senior authors (SH and MA). In all patients, the same surgical system with pedicle screws (additionally with hooks for proximal anchoring if needed) was used. Further inclusion criteria included the availability of full-spine radiographs in the anterior-posterior and lateral view pre- and postoperatively. Patients with congenital, neuromuscular and syndromic scoliosis were excluded. All data collection was performed by a singe reader (AM), a research fellow with a medical background. Only completely filled patients’ questionaries and properly performed radiographs were included in this study. Data collection was conducted at least two years postoperatively or later. Figure 1 illustrates the inclusion process. This study was approved by the ethics committee of Heidelberg University (permission No. S-748/2019).

### 2.2. Data Collection and Analysis

**Figure 2:**
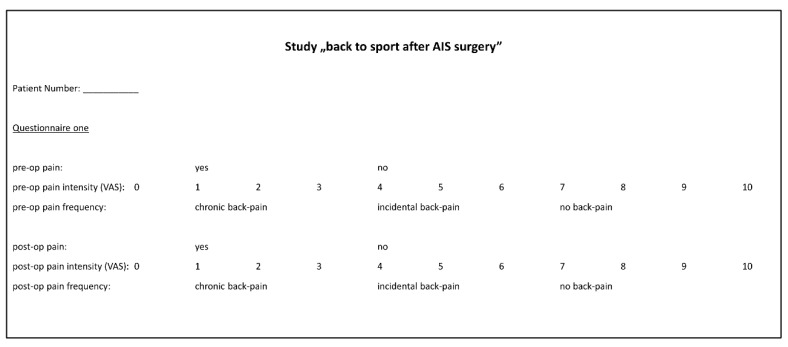

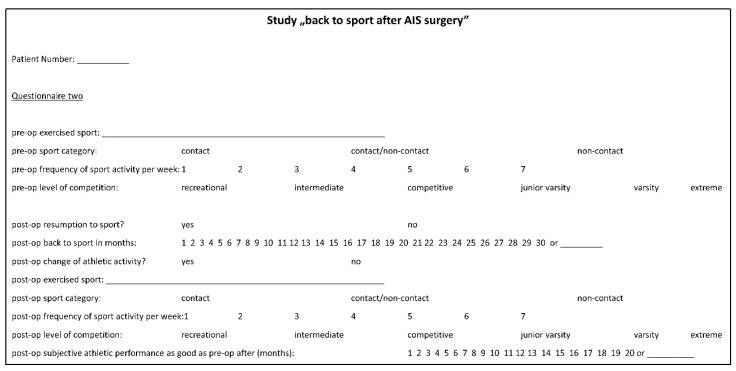
Questionnaires used for data acquisition.

For present study, to investigate athletic activities, AIS patients were enrolled. All participants gave informed consent (or a guardian provided consent for patients under 18 years). The study was based on two questionnaires assessing patients’ contentment and athletic activity pre- and postoperatively (Figure 2). Attempts were made for each patient who met the inclusion criteria to contact a study coordinator by phone. Patients were asked a series of questions in relation to returning to a daily routine and participating in physical and sporting activities. In addition, questions were asked in relation to their competition level before and after surgery. Simultaneously, parents of the study participants confirmed the statements. Data were recorded at the time of surgery and collected from medical records for all patients who met the inclusion criteria, including sex, age at the time of surgery, date of surgery, body mass index (BMI), preoperative and postoperative visual analog pain score (VAS) (Figure 2), instrumentation used (e.g., all pedicle screws, mixed construct), perioperative complications, time to clearance to participate in athletic activity and any athletic or physical activities that the patient was involved in before and after surgery. Patients were asked a series of questions regarding preoperative and postoperative participation in physical and athletic activities and their level of competition (e.g., recreational, travel, junior varsity, varsity). Finally, activities were categorized into three categories: (1) contact, (2) contact/non-contact and (3) non-contact (Table 1). Diverse preoperative sports with only one or two participants were acrobatics, ballet, dancing, skiing, basketball, boxing, bicycle riding, climbing, football, running, handball, swimming, dog sports, jogging, karate, running, athletics, paddling, rowing, skiing, tennis, table tennis, volleyball and yoga. Diverse postoperative sports with only one or two participants were acrobatics, climbing, jumping, yoga, coordination training, running, athletics, horse riding, karate, ski, gymnastics, volleyball and pilates.

### 2.3. Radiographic Assessment

**Figure 3:**
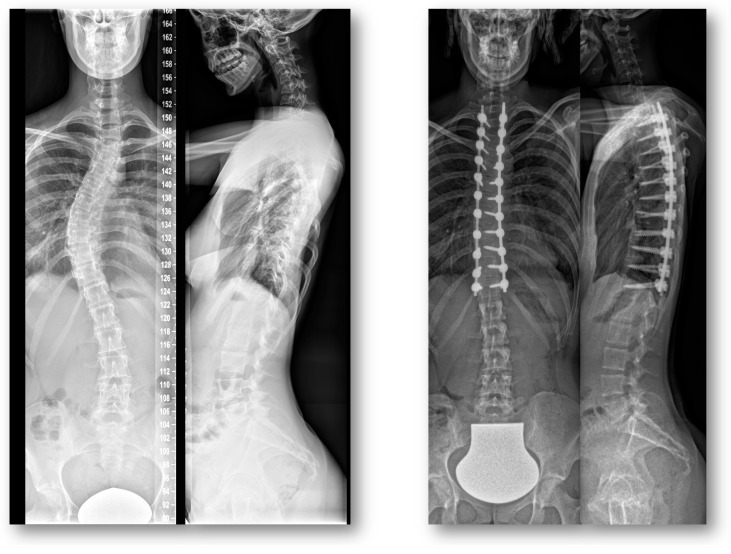
Pre-operative and post-operative coronal and lateral radiographs of study patients with adolescent idiopathic scoliosis and posterior fusion.

The radiographs performed for the imaging of the full spine before and after the posterior fusion were analyzed by a single reader (AM). Radiographic parameters included the following: evaluation of the fusion length, estimation of upper instrumented vertebra (UIV), estimation of lower instrumented vertebra (LIV), calculation of screw/pedicle index and rating based on the Lenke classification [19]. Preoperatively, the Cobb angle of the structural and compensatory curves was estimated (Figure 3).

### 2.4. Patient Stratification

According to the fusion length, the study population was divided into two groups: ≤ 10 fused levels and > 10 fused levels. Further stratification was performed due to the fusion location: thoracic fusion vs. thoracolumbar fusion. All patients’ characteristics and the pre- and postoperative athletic activities of both groups were compared.

### 2.5. Statistical Analysis

Software package SPSS^®^ Version 25 (IBM^®^, Armonk, NY, USA) was used for statistical analysis. Data were portrayed as mean and standard deviations. Intergroup comparisons were conducted using a paired *t*-test. Pearson’s test was used for correlation analysis. The threshold for statistical significance was set at *p* < 0.05. The absolute values of the coronal plane were utilized for recognition of the severity of deformity. This study was reviewed by a statistical consultant and study co-author (TB). After data collection and before statistical analysis, the data were pseudonymized.

## 3. Results

### 3.1. Global Analysis

A total of 113 patients (74.3% females, *n* = 84; 25.7% males, *n* = 29) with a mean age 16.4 ± 4.0 years (range: 10–22) were included in the study. Surgically treated patients had a weight 56.2 ± 14.8 kg and body height 164.3 ± 9.1 cm (body mass index, BMI: 20.6 ± 4.5).

In terms of the main (COBB 1) and secondary (COBB 2) scoliotic curve, at the time of indication for scoliosis correction, COBB 1 was at 53.4° ± 10.2° and COBB 2 at 34.1° ± 13.6°. Table 2 illustrates the distribution of scoliosis types within the study population based on the Lenke classification [19]. There was no significant correlation between these factors and the timepoint of return to athletic activities.

All patients were treated with a posterior procedure with correction surgery using pedicle screws. In addition, two patients were treated with hooks for proximal anchoring. In terms of the fusion level, most instrumentations were performed up to the L3 level (37.2%) and to the T3 level (29.6%) (Table 3). For the surgical exposition of the spine, 21.3 ± 5.9 pedicles were exposed for the possible implantation of the pedicle screws. For the implantation of screws, 17.6 ± 5.7 screws (minimum: 10 screws, maximum: 30 screws) were used for the correction maneuver. Therefore, the calculated screw index (implanted screws/exposed pedicles for possible screws implantation) was 0.8 ± 0.2.

### 3.2. Sport Activities and Returning to Sports after Surgery

**Figure 4:**
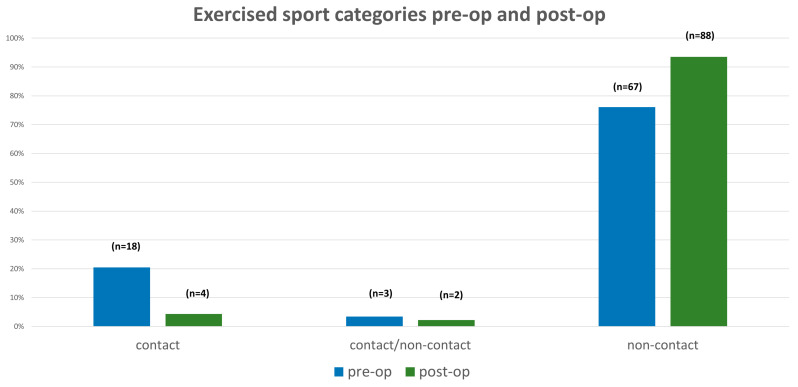
Diagram with an illustration of the exercised sports categories pre- and postoperatively.

Preoperatively, 88 patients (78% of the whole study population) were active in sports. Based on the sports categories, 18 patients (20.5%) practiced contact sports, 3 patients (3.4%) practiced contact/non-contact sports and 67 patients (76.1%) practiced non-contact sports. After surgery, the rate of patients with sports practice increased to 94 subjects (89%). Contact sports were postoperatively much less attractive for surgically treated patients. Only four patients (4.3%) practiced contact sports postoperatively. In addition, the rate of participation in contact/non-contact sports remained unchanged excluding two patients (2.2%). The group of patients who practiced non-contact sports increased to 88 patients (93.5%) (Figure 4). Postoperatively, 45% of the study population who were preoperatively active in sports changed their activities to other categories. Based on the practiced sports frequency per week, there was no significant change pre- and postoperatively (pre-op 1.7 ± 1.5 per week vs. post-op 1.9 ± 1.4 per week, *p* = 0.202). Generally, surgically treated scoliosis patients could return to sports activities after 8 months (back to sport: 8.0 ± 7.1 months after surgery). Due to their subjective athletic performance, the preoperative status could be achieved after 5 months (recovery of athletic performance to preoperative status after surgery: 5.4 ± 4.9 months). Further subanalysis revealed that 33 patients were able to return to exactly the same sport activities they had participated in preoperatively. These patients returned to their sport activities after 10 months (back to sport: 9.9 ± 6.7 months after surgery). In this subgroup, there was a slight difference between the non-contact and contact groups due to the time of return to sports postoperatively (back to sport non-contact vs. contact group: 9.7 ± 6.7 months vs. 11.0 ± 7.5 months).

Correlation analysis did not reveal any significant differences between preoperative back pain frequency (chronic pain vs. incidental pain vs. pain-free) and the time of returning to sports. The age of the patients at surgery time did not have any influence on the postoperative time of returning to sports (r = 0.057; *p* = 0.548). In addition, the sports category had no influence on the time of returning to athletic activities. Furthermore, studying the preoperative radiological status of scoliosis, we noticed that the preoperative severity of the primary scoliotic curve (Cobb angle) did not have any influence on the time of return to athletic activities (r = −0.052; *p* = 0.587).

### 3.3. Stratification by the Length and LIV of the Posterior Spondylodesis

We postulated that the fusion length could have an influence on the postoperative time of returning to sports. To analyze that, the study cohort was divided into two groups according to the length of the performed fusion. The first group (group I) contained 57 subjects with posterior spondylodesis performed in up to 10 segments. In the second group were included 56 patients with posterior spondylodesis in more than 10 segments. Both groups revealed no statistical differences based on the time of reaching the preoperative efficiency level (group I vs. group II: 5.06 months ± 5.02 vs. 5.89 months ± 4.79, *p* = 0.294) and time of returning to athletics (10.28 months ± 8.98 vs. 8.57 months ± 6.41, *p* = 0.384).

Furthermore, the LIV of the performed posterior fusion might have an influence on the time of returning to sports postoperatively. To analyze this hypothesis, the study group was divided into groups based on the location of the LIV of the performed fusion. The patients with the last instrumented vertebra in the lower thoracic spine were placed into one group (*n* = 20). Patients with the LIV at level L1 were *n* = 15, L2—*n* = 16, L3—*n* = 41 and L4—*n* = 19. Two patients with L5 due to a very small subgroup were excluded. The performed ANOVA test revealed no statistical differences of the tested subgroups regarding the time of returning to sports postoperatively (*p* = 0.418).

### 3.4. Clinical Outcome

**Figure 5:**
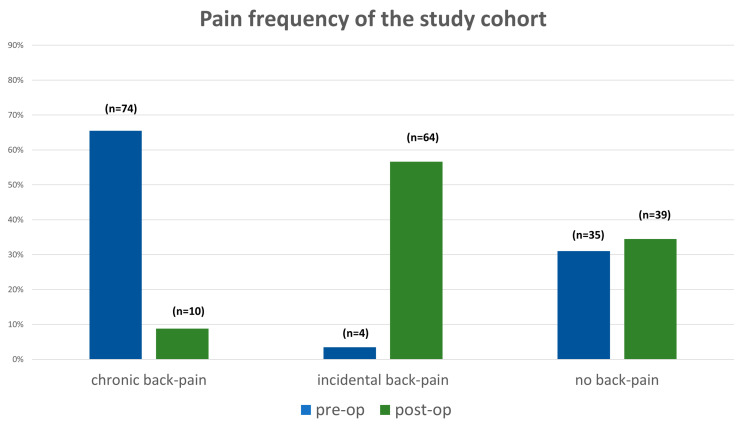
Diagram of back pain frequency pre- and postoperatively for the whole study population.

Preoperatively, a relevant part of the study population declared having chronic back-pain (74 patients of the whole study population). Postoperatively, the rate of patients with chronic back pain fell significantly to 10 patients. On the other hand, the rate of patients with incidental back pain raised postoperatively from 4 patients preoperatively to 64 patients postoperatively. The rate of patients with no back pain preoperatively (35 patients) increased insignificantly to 39 patients postoperatively (Figure 5). The study of pain intensity of the whole study population revealed a significant decrease when comparing pain intensity preoperatively to postoperatively (VAS pre vs. VAS post: 3.9 ± 3.2 vs. 3.1 ± 2.7, *p* = 0.009).

## 4. Discussion

Posterior correction with a fusion for the treatment of AIS patients remains mostly a prophylactic procedure, in that the goal of this therapy is the prevention of symptoms such as debilitating pain and disability in the future. Mainly, adolescents with scoliosis are as active in sports as their unimpaired peers. Surgical treatment of AIS patients leads to a temporary pause of activity in sports, which may be for the young patients’ benefit. For that, the issue of the postoperative pause recommendation for sports activity is a relevant subject in the daily clinical practice of spine surgeons. So far, consensus guidelines and postoperative timing for the return to sporting activities after AIS correction have not been established [20,21,22]. Most recommendations are still based on experts’ opinions and experts’ consensus.

Throughout recent decades, surgical AIS therapy has undergone meaningful improvement. First, surgical AIS therapies, e.g., Harrington instrumentation, were associated with complications and deficient primary stability [23,24]. Often, an additional brace or cast therapy after former surgical AIS therapy were obligatory, and sports activities were prohibited. The implementation of pedicle-screw systems in posterior fusion surgeries has led to the significant improvement in primary stability. Further development of the pedicle screw design improved surgical outcomes and the immediate postoperative stability in patients with multi-segmental constructs [25,26]. In modern surgical AIS therapy, due to the industrial improvement in spondylodesis implants, brace therapy is now not required. Concerning improvements in posterior stabilization systems, it should be discussed and analyzed with randomized and prospective control trials whether an earlier return to sports for surgically treated AIS patients would be possible without any complications. On the other hand, the planning and enforcement of such clinical trials, due to the unconformity to ethical rules, is almost infeasible. For that, data about recommendations to return to sports after AIS surgery are very sparse.

Based on experts’ general opinions, within the first post-surgical year, every sports activity should be considered and exercised with stringent carefulness and with former consultation with the surgeon. Within the first months and up to one year postoperatively, the aim of the implanted stabilization system is to reach a bony fusion, which should not be compromised by repetitive overstraining. Normally, if sufficient primary stability during the surgical procedure was reached, then bicycling could be allowed six months after spondylodesis [27]. Based on experts’ opinions, school sports participation is allowed one year after surgery and club sports is allowed after 24 months [27]. Contact sports with a higher potential of stress on the spine should be avoided [28]. Sports with a risk of uncontrolled falls, e.g., horse riding, are associated with a higher risk of serious injury in comparison to the controls without a posterior fusion [28]. Based on the postoperative ambulatory clinical controls in our department, we noticed a discrepancy between common recommendations and the certainty surrounding sports exercising. This study demonstrates the postoperative behavior of surgically treated AIS patients in a single center in terms of returning to sport activities.

In accordance with other investigations [29,30,31], this study population experienced pain relief that reached a level of significance. New in this research is the study of pain quantity and its changes from a pre- to postoperative status. In this study cohort, we noticed a relevant shift from chronic pain to occasional discomfort, indicating improved therapy outcomes. This condition might be an explanation for early patients’ recovery of body confidence and their earlier return to sports than recommended. Furthermore, a significant shift of club sports to spare time sports and a significant shift from contact sports to non-contact sports may also facilitate the earlier return to sport activities. In this study cohort, the average time of return to athletic activities was 8 months postoperatively, which is in line with the present conclusions of other groups [20]. Furthermore, we noticed a large range of time for returning to sport activities. Few patients who participated in non-contact sports were able to return to their sport activity very quickly; on the other hand, patients with contact sports achieved it at a later timepoint postoperatively. Nevertheless, in this study, there was no significant difference between both groups. On average, both groups (contact and non-contact) achieved their timepoint of return to athletic activities after 8 months.

Interestingly, further subanalysis of the study cohort revealed that 33 patients (29.2% of the whole study population) could return to exactly the same sport activity as before surgery. This subgroup could return to sports activities after 10 months. Within this subgroup, further analysis due to sport categories revealed just a slight difference due to a return to sports. The non-contact (*n* = 30) group returned to preoperatively exercised sports 1 month earlier than the contact group (*n* = 3). In this context, the performance of the statistical test for significance was not possible due to the too-small contact sports subgroup (*n* = 3).

In contrast to our hypothesis, there was no correlation between the length of the performed fusion and the time of returning to athletic activity. Furthermore, we performed the LIV stratification to disentangle the possible influence of the LIV on the time of returning to athletic activities. Our results are in contrast to the results of Fabricant et al. [20]. In their research, the relationship between the distal level of the spinal fusion and the percentage of patients who returned to athletics at the same or higher level of competition with an odds ratio of 0.633 and level of significance (*p* = 0.039) could be clearly delineated. These results may be explained by a higher sensitivity of data acquisition using a validated SRS-22 score [32] in comparison to the data acquisition in the present study. On the other hand, the explanatory power of the statistical analysis of 42 subjects in the presented study cohort might be limited.

The main limitation of this study is inherent to its retrospective design and absence of a control group. There was no control group in this study to determine the natural transformation of adolescents due to athletic habits. Second, ethical considerations about different recommendations for the prospective subgrouping of the study cohort due to the time of postoperative return to athletic activities, which might be associated with possible mechanical complications, is prohibited. Furthermore, the explanatory power of this study due to heterogeneous conditions and the small study cohort is restricted. However, this study provides valuable clinically important information regarding the time of returning to athletic participation after AIS surgery. The postoperative return to athletics is multifactorial. Therefore, it is difficult to postulate why certain associations with clinical and surgical variables exist. Prospective studies are needed to precisely determine the time required to return to athletics and for a better understanding of the possible variables that have an influence on postoperative sports habits.

## 5. Conclusions

On average, AIS patients returned to sport activities 8 months postoperatively. A total of 29.2% of the study population (*n* = 33) could return to exactly the same sport activity as preoperatively. In this subgroup, a return to sporting activities took 10 months of postoperative rest. This study revealed a postoperative shift from contact sports to non-contact sport activities. Interestingly, the length of the performed posterior fusion and fusions to the lower lumbar spine had no influence on the time of return to athletic activities. These results might be useful for surgeons’ pre-treatment consultations when informing AIS patients about postsurgical outcomes. Returning to sports seems to be achieved earlier than prevalent surgeons’ recommendations. It is important to understand that this study analyzed patient trends in returning to sports and cannot be considered as a guideline in making specific treatment recommendations. At this stage, definitive recommendations for the return to sporting activities after a posterior fusion in AIS patients should be made based on each patient’s expectations, goals and past experiences.

## Figures and Tables

**Table 1:** Sport categories of the study cohort.

Pre-Op Contact	Pre-Op Contact/ Non-Contact	Pre-Op Non-Contact	Post-Op Contact	Post-Op Contact/ Non-Contact	Post-Op Non-Contact
		Bicycle (*n* = 5)			Bicycle (*n* = 7)
		Fitness (*n* = 6)			Fitness (*n* = 37)
Football (*n* = 11)			Football (*n* = 4)		
		Horse riding (*n* = 5)			
					Jogging (*n* = 7)
	School sports (*n* = 12)			School sports (*n* = 6)	
		Swimming (*n* = 7)			Swimming (*n* = 13)
		Dancing (*n* = 14)			Dancing (*n*= 7)
					Walking (*n* = 5)
		Gymnastics (*n* = 4)			

**Table 2:** Distribution of the different types of adolescent idiopathic scoliosis patients based on established Lenke classification [14].

Curve Type	Quantity	Percentage
Main thoracic (Type 1)	35	31.0%
Double thoracic (Type 2)	28	24.8%
Double major (Type 3)	20	17.7%
Triple major (Type 4)	7	6.2%
Thoracolumbar/lumbar (Type 5)	7	6.2%
Thoracolumbar–main thoracic (Type 6)	16	14.1%
Total	113	100.0%

**Table 3:** Distribution of the upper instrumented levels (UIV) and lower instrumented levels (LIV) of the study population.

UIV	Quantity	Percentage	LIV	Quantity	Percentage
T2	18	15.9%			
T3	33	29.6%	T10	2	1.8%
T4	27	23.3%	T12	18	15.9%
T5	8	7.2%	L1	15	12.2%
T6	8	7.2%	L2	16	14.1%
T7	2	1.8%	L3	41	37.2%
T9	2	1.8%	L4	19	17%
T10	12	10.5%	L5	2	1.8%
T11	2	1.8%			
T12	1	0.9%			
Total	113	100.0%	Total	113	100.0%

## Data Availability

The datasets used and analyzed during the current study are available from the corresponding author on reasonable request.
